# Gulonolactone Addition to Human Hepatocellular Carcinoma Cells with Gene Transfer of Gulonolactone Oxidase Restores Ascorbate Biosynthesis and Reduces Hypoxia Inducible Factor 1

**DOI:** 10.3390/biomedicines2010098

**Published:** 2014-03-05

**Authors:** Teresa Flett, Elizabeth J. Campbell, Elisabeth Phillips, Margreet C. M. Vissers, Gabi U. Dachs

**Affiliations:** 1 Mackenzie Cancer Research Group, Department of Pathology, University of Otago, PO Box 4345, Christchurch 8140, New Zealand; E-Mails: flete688@student.otago.ac.nz (T.F.); elizabeth.campbell@otago.ac.nz (E.J.C.); elisabeth.phillips@otago.ac.nz (E.P.); 2 Centre for Free Radical Research, Department of Pathology, University of Otago, PO Box 4345, Christchurch 8140, New Zealand; E-Mail: margreet.vissers@otago.ac.nz

**Keywords:** vitamin C, HIF-1, gene therapy

## Abstract

Humans are unable to synthesise ascorbate (Vitamin C) due to the lack of a functional gulonolactone oxidase (Gulo), the enzyme that catalyses the final step in the biosynthesis pathway. Ascorbate is a vital micronutrient required for many biological functions, including as a cofactor for metalloenzymes that regulate the transcription factor hypoxia-inducible factor-1 (HIF-1), which governs cell survival under hypoxia. In most animals, ascorbate is made in liver cells. This study aimed to restore ascorbate synthesis to human hepatocellular carcinoma HepG2 cells and determine the effect of internally produced ascorbate on HIF-1 activation. HepG2 cells were gene-modified with a plasmid encoding the mouse *Gulo* cDNA, tested for genomic incorporation by PCR and ascorbate synthesis by high performance liquid chromatography. Levels of HIF-1 protein were measured using Western blotting. Gulo-modified HepG2 cells showed increased adherence compared to control HepG2 cells. A PCR-positive clone synthesised ascorbate when the Gulo substrate, l-gulono-1,4-lactone, was supplied. Intracellular ascorbate concentrations reached 5% of saturation levels (6 nmol/10^6^ cells). Addition of ascorbate or gulonolactone reduced HIF-1 accumulation in the Gulo clone, but also in parental HepG2 cells. Our data confirm the requirement for a number of factors in addition to Gulo in the ascorbate biosynthesis pathway in human cells.

## 1. Introduction

Ascorbate is a vital micronutrient required for many biological functions, including its action as a potent antioxidant and cofactor for metalloenzymes [1]. As a cofactor for prolyl- and asparaginyl-hydroxylase (Fe(II) and 2-oxoglutarate-dependent dioxygenases), it controls degradation and transcriptional activity of hypoxia inducible factor-1 (HIF-1) (reviewed by [2]). 

HIF-1 is the master regulator of molecular responses to low oxygen (hypoxia) in cancer, as it regulates tumour growth and spread via transcriptional regulation of >100 genes involved in glycolysis, angiogenesis and cell survival [3]. A lack of HIF-1 resulted in reduced tumour growth and angiogenesis in an animal model [4]. In cancer patients, over-expression of HIF-1 is associated with tumour progression, resistance to therapy and poor prognosis (reviewed by [5]). HIF-1 inhibition has been suggested as a therapeutic strategy in cancer and HIF-1α is actively being validated as a drug target in clinical trials [6]. 

Ascorbate levels in tumours from cancer patients have been measured directly [7,8]. Data showed that high grade endometrial [7] and colorectal [8] tumours accumulated less ascorbate than low grade tumours. Similarly, large tumours contained significantly less ascorbate than smaller ones. A striking link was demonstrated between low tumour ascorbate levels, increased HIF-pathway related proteins, and tumour aggression [7,8]. 

Optimal supplementation of cancer cells in tissue culture with up to 1 mM ascorbate can suppress HIF-1α induction by a range of stimuli, and can reduce indicators of cancer aggression (VEGF-A, Glut-1, and BNIP3) [9]. However, due to the abnormal nature of the tumour vasculature [10], regions of tissue may not receive adequate ascorbate even if plasma levels were optimal.

Ascorbate also plays a major role in the synthesis of collagen, and collagen fibers form part of the extracellular matrix. *In vitro*, collagen increased directional movement of tumour cells, and introduction of ascorbate into the media reduced this metastatic phenotype [11].

Humans are among a handful of animals unable to synthesise their own ascorbate, as we lack the final enzyme in the ascorbate biosynthesis pathway, gulono lactone oxidase (Gulo) [12,13]. Gulo converts l-gulono-1,4-lactone (gulonolactone) to l-ascorbic acid (ascorbate) in the liver of most animals. Plasma levels of ascorbate are tightly controlled via intestinal uptake and renal clearance, cellular levels are regulated via ascorbate transporters, and ascorbate is labile and short-lived [14]. 

The aim of this study was to determine whether it was possible to restore ascorbate biosynthesis to human liver cancer cells by transfer of the mouse Gulo-encoding gene, and to determine the impact of intracellular ascorbate on HIF-1 levels.

## 2. Results and Discussion

### 2.1. Transfection

**Figure 1:**
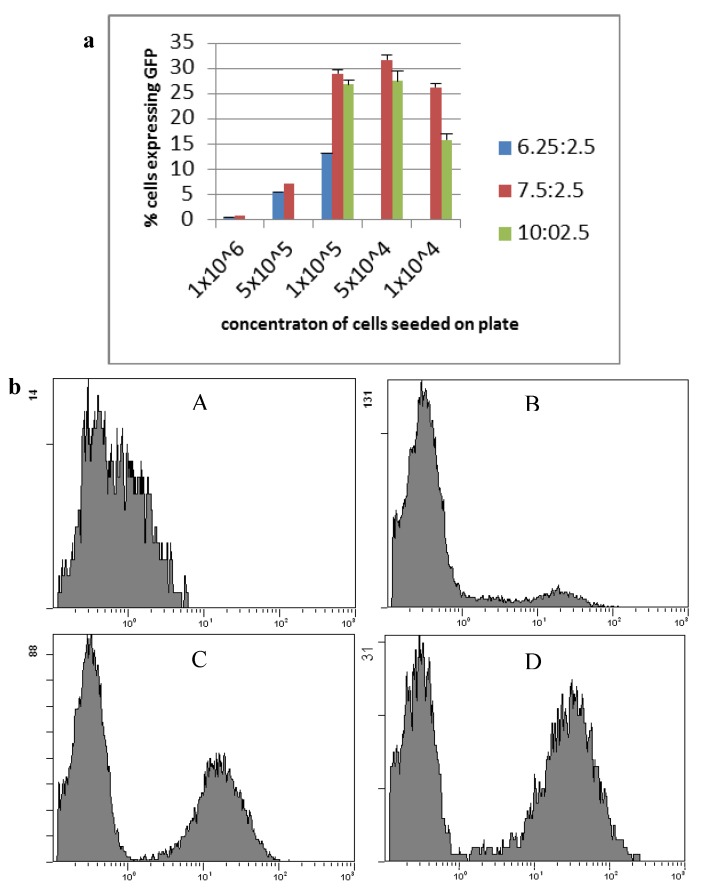
Transfection of HepG2 cells using lipofectamine LTX and pcDNA3-EGFP. (**a**) HepG2 cell line transfection optimisation of lipofectamine LTX ratio and cell seeding concentrations counted by the average number of cells expressing GFP. **Blue**: a ratio of 6.25 µL lipofectamine LTX to 2.5 µg plasmid; **Red**: a ratio of 7.5 µg lipofectamine LTX to 2.5 µg plasmid; **Green**: a ratio of 10 µL lipofectamine LTX to 2.5 µg plasmid (*n* ≥ 3, mean ± standard deviation); (**b**) Flow cytometry of HepG2 cells expressing GFP. (**A**) HepG2 parental cells; (**B**) HepG2 GFPB1 cells; (**C**) HepG2 GFPC2 cells; (**D**) HepG2 GFPC1 cells.

Transfection of HepG2 cells was optimised using the reporter plasmid pcDNA3-EGFP and subsequent fluorescent activated cell sorting (FACS) (Figure 1). A ratio of 7.5:2.5 lipofectamine LTX to plasmid with a seeding density of 1 × 10^5^ cells per well gave the highest transfection efficiency, with lowest toxicity, and was used for future transfections. This protocol resulted on average in 29% ± 4.9% of cells being gene modified. Following selection on G418 for over 50 days resulted in 53% of transfected cells expressing GFP, and selection for over 100 days resulted in 71% of cells expressing GFP (clone HepG2 GFPC1).

### 2.2. Cell Morphology

**Figure 2:**
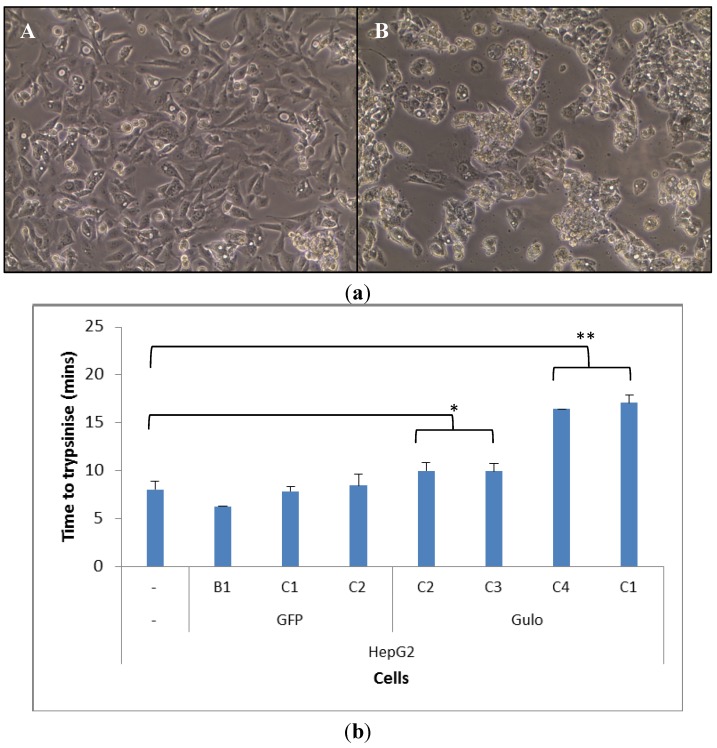
Morphology and adhesion of transfected HepG2 cells. (**a**) Microscopic view of HepG2 (**A**) and HepG2 GuloC1 (**B**) cells (200× magnification); (**b**) Time for trypsinisation of HepG2 cell lines; *n* = 5, mean ± SD, *t*­test (two tailed unpaired) (* *p* < 0.05, ** *p* < 0.01).

Once HepG2 cells were transfected they took on a different morphology, particularly the HepG2 GuloC1. The transfected cells grew on top of each other, which was less evident in the parental HepG2 cells, on occasions resulting in the formation of spheroids blebbing off the plate surface (Figure 2a). The HepG2 GuloC1 clone took a significantly longer time to trypsinise off the plate than its parental HepG2 counterpart (*p* < 0.01, Figure 2b). This was also seen in three other pCMV-Gulo-modified HepG2 cultures which were under selection pressure for shorter periods (*p* < 0.05). Cells that were transfected with pcDNA3-EGFP rather than pCMV-Gulo did not show this effect (Figure 2b). The morphological changes observed in all Gulo-modified HepG2 cells, but not in any EGFP-modified cells, indicate that a biologically active factor was produced. 

### 2.3. Genomic Insertion

**Figure 3:**
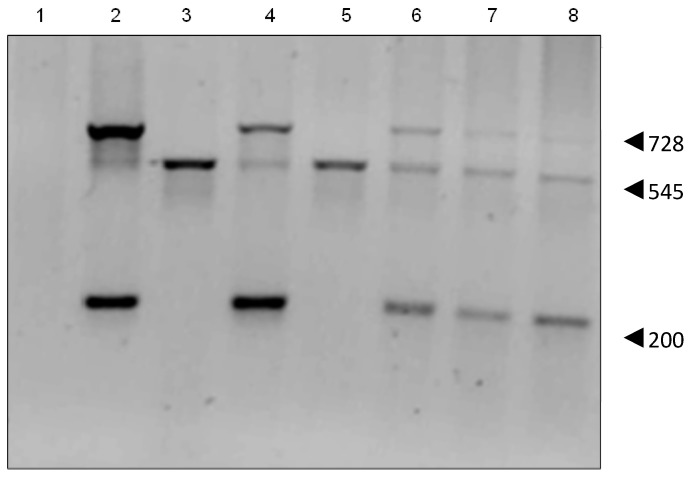
PCR of HepG2 transfectants. The 545 bp product indicates human pseudo *Gulo* gene, the 200 bp and 728 bp products the mouse cDNA *Gulo* gene. Lanes **1**: PCR reaction using H_2_O; **2**: pCMV6–Gulo pure plasmid; **3**: HepG2; **4**: HepG2 GuloA1; **5**: HepG2 GuloB1; **6**: HepG2 GuloC1; **7**: HepG2 GuloC2; **8**: HepG2 GuloC3 (all products are pooled PCR reactions of primer pairs 1&2, 1&5, 3&4).

PCR analysis confirmed that the pCMV6-Gulo plasmid contained the mouse cDNA for Gulo, showing the expected 200 bp and 728 bp products. HepG2 cells showed a 545 bp product confirming that they contain the human pseudo gene (*NPT II*). PCR of the HepG2 transient transfectants showed both large and small Gulo PCR products (Figure 3). Each of the stable HepG2 GuloC clones showed the presence of both the human pseudo gene and the mouse cDNA *Gulo* gene. 

### 2.4. Ascorbate Synthesis

**Figure 4:**
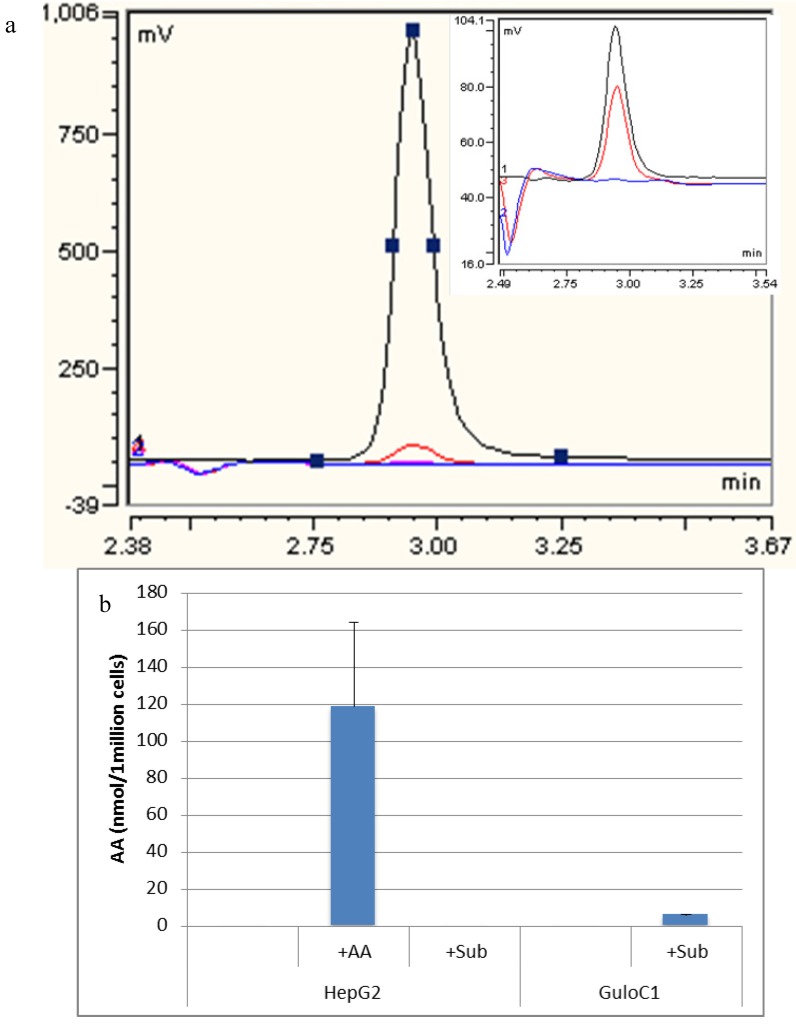
Detection of ascorbate by HPLC. (**a**) Representative HPLC-ECD traces of assessment of HepG2 GuloC1. **Blue**: HepG2; **Black**: HepG2 + 500 µM ascorbate diluted 1/10; **Red**: HepG2 GuloC1 + 500 µM gulonolactone; **Pink**: 500 µM gulonolactone. Insert: **Blue**: HepG2; **Black**: ascorbate 2.5 µM standard; **Red**: HepG2 GuloC1 + 500 µM gulonolactone; (**b**) Amount of ascorbate detected by HPLC-ECD in HepG2 parental and HepG2 GuloC1 cells. +AA = 500 µM ascorbate added, +Sub = 500 µM gulonolactone added (*n* = 3, mean ascorbate per 1 million cells ± SD).

High performance liquid chromatography (HPLC) analysis detected no ascorbate in the growth medium or in the parental HepG2 cell line. When 500 µM of ascorbate was added to the media, HepG2 cells accumulated a mean of 118.6 nmol (±79) per 1 million cells over 24 h (Figure 4). Despite the presence of the mouse Gulo-encoding gene (by PCR), no intracellular ascorbate was detected in GuloC1 clones or any of the Gulo-modified HepG2 cells. No ascorbate was detected in HepG2 parental cells when up to 500 µM of gulonolactone was added. When 500 µM of gulonolactone was added to the HepG2 GuloC1 clone, they produced 6 nmol/10^6^ cells ascorbate. A small peak by HPLC was evident, equivalent to the 2.5 µM (0.05 nmol/20 μL) standard (Figure 4). This represents about 5% of the optimal amount accumulated following extracellular addition of 500 µM of ascorbate. Published data, using virally modified HepG2 cells, reported production of 50 nmol/10^6^ cells following treatment with 10 mM of gulonolactone [15,16].

### 2.5. Hypoxia Inducible Factor 1 Levels

**Figure 5:**
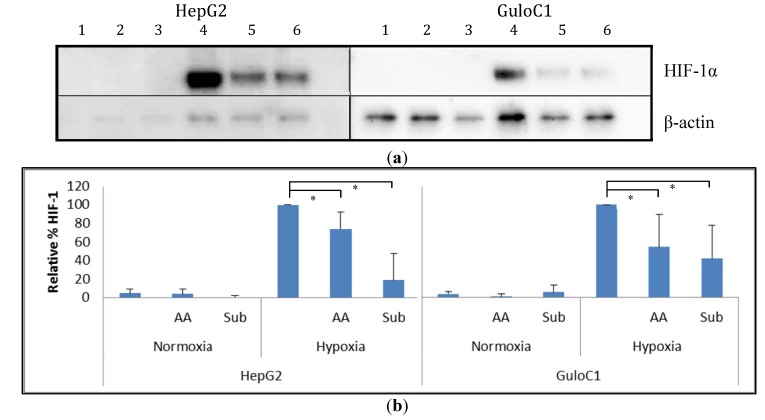
Western blot analysis of HIF-1α protein in HepG2 parental and HepG2 GuloC1 cells. (**a**) Representative image of a Western blot of cells exposed to air (1–3) or hypoxia (4–6), with no additions (1, 4), with 500 µM of ascorbate (2, 5) or 500 µM gulonolactone (3, 6); HIF-1α was detected as a 120 kDa protein, and β-actin was used to standardise loading; (**b**) Estimation of HIF-1α protein levels from Western blots by densitometry. Means ± SD from *n* = 4 Western blots are shown, relative to HIF-1α levels in hypoxic cells without additional treatment, standardised to β-actin; ascorbate, AA, gulonolactone, Sub; * *p* < 0.05.

Under normoxia, minimal basal HIF-1α protein levels were detected in parental HepG2 cells. Exposure to hypoxia (1% O_2_) increased HIF-1α protein levels, and the addition of 500 µM of ascorbate reduced these levels (Figure 5). Similarly, HepG2 GuloC1 cells had minimal detectable HIF-1α protein in air. Under hypoxia, the HepG2 GuloC1 cells showed increased levels of HIF-1α and again the level was reduced with the addition of ascorbate. When 500 µM of gulonolactone was added to hypoxic HepG2 GuloC1 cells, a similar reduction in HIF-1α was evident (Figure 5). Addition of gulonolactone also reduced hypoxic HIF-1α protein in HepG2 parental cells. 

## 3. Experimental Section

### 3.1. Cells and Cell Culture

The epithelial hepatocellular carcinoma cell line HepG2 (HB 8065) was purchased from the American Type Culture Collection. Cells were maintained in DMEM GlutaMax (Gibco, Auckland, New Zealand) with 10% Fetal Bovine Serum (Invitrogen, Auckland, New Zealand) in a humidified 5% CO_2_, 20% O_2_ at 37 °C Heraeus incubator. Cells were passaged using TrypLE Express (Gibco) and reseeded at 30% confluence once a week.

### 3.2. Hypoxic Conditions

Hypoxic cells were incubated for 24 h in a Whitley H35 Hypoxystation under conditions of 1% O_2_, 5% CO_2_, balanced with N_2_. Media, plastics and supplements were left in the H35 for 3 h to equilibrate prior to addition to cells.

### 3.3. Transfection

Murine Gulo cDNA (NM_178747) inserted into a pCMV6-Kan/Neo plasmid was obtained from Origene Technologies. pEGFP-N1 from Clontech was used in parallel as control. Plasmids were purified using the Endo-free plasmid Maxi kit (Qiagen, Melbourne, Australia) following manufacturer’s protocol. Following optimisation using a range of ratios and cell numbers, HepG2 transfections were carried out using a ratio of Lipofectamine LTX (Invitrogen, Auckland, New Zealand) to plasmid of 6.25:2.5 at a cell density of 1 × 10^5^ cells per well in a six-well plate. Transfection efficiency was measured in HepG2 cells transfected with pEGFP-N1 using the Cytomics Fc500 flow cytometer to detect green fluorescent protein. A series of transfectants was generated which differed in the time since transfection: GFPA and GuloA denoted transient transfectants within 48 h of transfection, GFPB and GuloB were enriched, mixed cultures which were placed under G418 selection pressure for up to two weeks, and GFPC and GuloC clones were picked as separate colonies from plates and expanded over 50–100 days under G418 selection. It was assumed that parallel transfections using the same protocol as with pEGFP-N1 with pCMV-Gulo resulted in a similar level of gene modification. HepG2 cells gene modified with pcDNA3-EGFP were used throughout as negative controls for pCMV6-Gulo modified cells.

### 3.4. PCR Analysis

Cells were lysed with Direct PCR Lysis Reagent (Viagen Biotech Inc., Los Angeles, CA, USA) with 0.1 mg/mL Proteinase K (Sigma-Aldrich, St. Louis, MO, USA) added following manufacturer’s protocol. Custom primers were designed from published sequences of the human pseudogene [17] and the mouse *Gulo* gene (Gene ID: 268756, [18]) and manufactured by Integrated DNA Technologies. *Gulo1*_F (TGGATCAACCGCTTCTTCTT) and *Gulo2*_R (GGGTAGTGGGCTACCACCTT) were used to produce the 200 bp *Gulo* cDNA product, *Gulo1*_F and *Gulo5*_R (ATGGCTCGTGGAGATGCAAA) were used to produce the 728 bp Gulo cDNA product, and *Gulo3*_F (TGTGCCTGTGTCTGTAGAGC) and *Gulo4*_R (AGACGCTGAATCCTCCCAAC) were used to produce the 545 bp human pseudo Gulo product. Platinum^®^ supermix was added to primers and sample DNA as by the manufacturer’s instructions. Samples were run with a 4 min hot start and then 35 cycles of 95 °C, 53 °C, then 70 °C for two and a half hours. BlueJuice™ Gel loading buffer (Invitrogen, Auckland, New Zealand) 10× loading buffer was added to samples and samples were separated run on a 2% SeaKem^®^ LE Agarose (Lonza, Auckland, New Zealand) gel containing SYBR^®^ safe DNA gel stain (Invitrogen) alongside a 100 bp ladder (Invitrogen). Bands were detected with UV fluorescence. 

### 3.5. Western Blot Analysis

Western blot analysis was performed following standard protocols. Briefly, 10 μg of total protein per lane was loaded to detect HIF-1α. Proteins were separated on a Bolt™ 4%–12% Bis–Tris Plus 17 well gels (Novex, Auckland, New Zealand), transferred to PVDF (Invitrogen, Auckland, New Zealand) membrane and blocked with 4%–5% milk in TBS-T (Sigma-Aldrich, St. Louis, MO, USA) for 1 h. Proteins were detected with rabbit polyclonal anti-HIF-1 (R&D Systems, Minneapolis, MN, USA, 1/800 in 4% milk overnight) and mouse monoclonal anti-β actin (R&D, 1/10,000 in 4% milk for 1 h. Primary antibodies were detected using rabbit anti goat HRP (Dako, Glostrup, Denmark, 1/10,000 for 45 min), and goat anti mouse HRP (Dako, 1/1000 for 30 min). Chemiluminescent bands were quantified using Alliance 2.7 software, and the protein of interest normalized against β-actin.

### 3.6. Ascorbate and Gulonolactone Loading

Solutions of 100 mM sodium l-ascorbate (Sigma-Aldrich, St. Louis, MO, USA) and 200 mM l-gulono-1,4-lactone (synonym gulonolactone, Sigma-Aldrich) were made up fresh in phosphate buffered saline and filter sterilized with 0.22 µm filters. The appropriate amounts were then added to fresh DMEM media. 

### 3.7. HPLC Analysis

Ascorbate concentrations were measured by HPLC with electrochemical detection as described before [9,19]. Briefly, trypsinised, viable cells were counted, washed in PBS and 1:1 0.54 M perchloric acid to qH_2_O was added. A small sample of supernatant was used to determine total protein. Samples were incubated on ice for 10 min and spun to remove cellular debris. The supernatant was stored at −80 °C. Samples for HPLC-ECD were diluted 1/10 with 1:1 ratio of 0.54 M PCA and qH_2_O and were kept on ice. A standard of ascorbate was produced using soldium l-ascorbate (Sigma-Aldrich, St. Louis, MO, USA) at 50 µM in 0.74 M PCA which was validated by measuring the absorbance at 254 nm. Dilution series containing fresh ascorbate (40 µM, 20 µM, 10 µM, 5 µM, 2.5 µM, 1.25 µM and 0 µM) or gulonolactone (500 µM, 100 µM, 50 µM, 10 µM and 0 µM) were made and run with each assay. The samples were analysed on a Waters™ 600 Controller HPLC with a Synergi 4u Hydro-RP 80A (Phenomenex^®^, San Jose, CA, USA) column. The mobile phase was acetate buffer (80 mM sodium acetate and 0.54 mM DTPA) with 170 μL/L octylamine (Sigma-Aldrich). Detection of ascorbate was performed by SciTech ESA Coulochem II ECD and analysed using Chromeleon 6 software (Dionex, Sunnyvale, CA, USA).

### 3.8. Statistical Analysis

Data are expressed as means and standard deviation. Data were analyzed using MS Excel 2010. The student’s two-tailed, unpaired *t*-test determined differences in means with α = 0.05 or less being significant.

## 4. Conclusions

The development of a genetically modified hepatic cell line has resulted in a human cell line that can produce its own ascorbate, but only when gulonolactone was supplied. This data, together with previous publications [15,16], indicate that the ascorbate biosynthesis pathway in human cells has been significantly modified and may contain numerous non-functional members besides gulonolactone oxidase. Future biochemical studies will determine which other enzymes in the pathway are non-functional.

The Gulo-modified clone demonstrated morphological changes and increased adherence. Ascorbate is an important cofactor for the production of collagen fibers that form part of the extracellular matrix [11,20]. These cells showed significantly greater adherence to the flask suggesting that collagen production may have been modified by *Gulo*-gene transfer.

Ascorbate and gulonolactone, at similar concentrations, were able to reduce hypoxia-induced HIF-1α protein levels in both Gulo-modified and parental HepG2 cells. This indicated a role for gulonolactone in hydroxylase-targeted HIF-1 degradation. Yet, a previous study, using purified proteins in a cell-free system, demonstrated that gulonolactone did not promote prolyl-hydroxylase activity [21]. It therefore appears that the reduction in HIF-1 levels may be due to unrelated metabolic effects of gulonolactone, although direct or indirect promotion of hydroxylase activity by gulonolactone in cells cannot be ruled out.

This study confirmed that gene transfer of the mouse gulonolactone oxidase encoding gene was insufficient in itself to restore ascorbate synthesis to human cells. Initial evidence is provided that the ascorbate precursor, gulonolactone, may dampen the HIF-1 response in HepG2 cells. 
